# The Dual Roles of Extracellular Vesicle Subtypes in Regulating Traumatic Brain Injury

**DOI:** 10.3390/ijms27125322

**Published:** 2026-06-12

**Authors:** Xu Zhang, Chao Zhou, Yun Xu

**Affiliations:** 1 Nanjing Drum Tower Hospital, Chinese Academy of Medical Sciences & Peking Union Medical College, Nanjing 210008, China; s2024039005@student.pumc.edu.cn; 2 Department of Neurology, Nanjing Drum Tower Hospital, Affiliated Hospital of Medical School, Nanjing University, Nanjing 210008, China

**Keywords:** extracellular vesicle, Extracellular Vesicle Subtypes, traumatic brain injury, neuroinflammation

## Abstract

Traumatic brain injury (TBI) is a global public health problem which causes long-term neurologic damage caused by both primary mechanical injury and secondary pathological processes. Extracellular vesicles (EVs) such as exosomes, microvesicles (MVs) and apoptotic bodies (ApoBDs) serve as critical vehicles mediating intercellular communication in the central nervous system (CNS) following TBI. The biogenesis and the content of EVs, including proteins, lipids and RNAs, are greatly changed and involved in the evolution of inflammation or tissue repairing after TBI. In this overview, we recapitulate the cellular origin of EVs and the function of EVs in the neuroinflammatory process after TBI, highlighting the dual regulatory roles of EVs in the biological response to TBI, whereby certain EV populations amplify secondary injury cascades, while others promote endogenous repair and recovery processes. We next investigate the progress in EV engineering and targeted delivery systems and report the potential mechanisms, emphasize the prospects and potential of engineered EVs for therapy, and comment on challenges and perspectives for clinical application in TBI.

## 1. Introduction

Traumatic brain injury (TBI) represents a significant global public health challenge, affecting millions of individuals each year. TBI encompasses a spectrum of conditions directly caused by external mechanical forces, including blunt impact, rapid acceleration/deceleration, and penetrating wounds [1]. Generally, the pathological consequences of TBI evolve through two primary stages: an acute injury phase followed by chronic neurodegenerative processes [2]. Following neurotrauma, in the absence of secondary injury mechanisms, primary mechanical damage occurs to neurons, glia, and blood vessels of the brain. The primary injury triggers a secondary neuroinflammatory cascade—involving excitotoxicity, oxidative stress, and sustained inflammation [3,4,5,6]—which amplifies initial damage and drives chronic neurodegeneration, ultimately culminating in persistent cognitive, emotional, and motor impairments [7,8,9]. Rising evidence indicates that neuroinflammation, driven primarily by the activation of glial cells (microglia and astrocytes), plays a pivotal role in both acute and chronic TBI [5,10,11]. This process involves the release of damage-associated molecular patterns (DAMPs), which activate immune responses and exacerbate neuronal dysfunction [12,13].

Extracellular vesicles (EVs), small lipid bilayer-bound particles secreted by cells, are now recognized as essential mediators of intercellular signaling [14]. The view of EVs has evolved from waste-disposal “trash bags” to pivotal regulators, actively regulating a wide spectrum of biological and pathological processes, including those within the central nervous system (CNS). By transporting a wide array of bioactive molecules (proteins, lipids, RNAs and even DNA fragments), EVs actively regulate critical processes in the CNS. They have been shown to influence neuronal communication, glial activation, and immune responses in both physiological and disease states [15]. In the context of TBI, EVs act as key mediators of the neuroimmune response by modulating inflammation, neuronal survival, and repair processes through their specific molecular cargo [16]. EVs can exert diametrically opposing effects—attenuating neuroinflammation and promoting neuronal survival in certain contexts, while propagating pathological proteins and amplifying inflammatory cascades in others. This apparent paradox is resolved by recognizing that EV function is exquisitely context-dependent, governed by an interplay of factors, including their biogenetic origin, the identity and activation state of the parent cell, their specific molecular cargo, the time after injury, and the characteristics of the recipient microenvironment.

Following TBI, extracellular vesicles (EVs), such as exosomes, microvesicles (MVs), and apoptotic bodies (ApoBDs), arise from distinct cellular processes and carry unique molecular signatures [17,18]. This functional diversity explains the complex roles EVs play in TBI. Consequently, elucidating their biogenesis, cargo, and cellular origins is critical for harnessing their potential as diagnostic biomarkers and therapeutic agents. Additionally, it is important to acknowledge that the operational distinction between exosomes, microvesicles, and apoptotic bodies is not always achievable with complete specificity in experimental studies. Most commonly used isolation methods—including differential ultracentrifugation, size-exclusion chromatography, and polymer-based precipitation—yield EV preparations that are enriched for a particular size range rather than for a single biogenetically defined subtype [18,19]. Consequently, functional effects attributed to “exosomes” in the literature may, in many cases, reflect the activity of mixed small EV populations, while “microvesicle” preparations can overlap with larger exosomes or small apoptotic bodies. In this review, we retain the subtype nomenclature as reported in the original studies for consistency; however, readers should interpret subtype-specific claims with caution, recognizing that the cargo and functions described may be characteristic of a broader class of EVs rather than exclusively linked to a single biogenesis pathway. Where possible, we indicate the isolation method employed in the cited studies.

Overall, we aim to elucidate the complex and dual roles of EV subtypes in TBI in this review. We first provide an overview of EV biogenesis and classification, establishing the basis for understanding their functional heterogeneity. Next, we dissect the cellular origins of EVs in the injured brain, highlighting how parent cell identity dictates vesicle cargo and function. Building on this, we comprehensively analyze the dichotomous effects of exosomes, microvesicles, and apoptotic bodies in both mitigating and exacerbating neuroinflammatory cascades, neuronal death, and oxidative stress. We then explore the translational frontier by discussing strategies for engineering EVs for targeted therapy and for achieving subtype-specific clearance, before finally addressing the critical challenges and emerging interdisciplinary approaches that will shape the clinical translation of EV-based diagnostics and therapeutics for TBI.

## 2. Methods

### 2.1. Literature Retrieval Databases

Three mainstream authoritative biomedical academic databases were selected for comprehensive literature retrieval, namely, PubMed, Web of Science Core Collection, and Embase. These databases cover high-quality preclinical and clinical studies in the fields of neuroscience, molecular biology, and regenerative medicine, enabling comprehensive collection of studies focusing on EV biogenesis, subtype classification, neuroinflammatory regulation, oxidative stress modulation, and engineered EV therapy in the context of TBI, thereby guaranteeing the comprehensiveness and academic authority of the research data included in this review.

### 2.2. Retrieval Keywords and Strategies

Retrieval keywords were formulated based on the core research themes of this review, combining subject terms and free terms to expand the retrieval scope and avoid omitting relevant literature. The English retrieval keywords included: Traumatic Brain Injury, TBI, Extracellular Vesicles, EVs, Exosomes, Microvesicles, MVs, Apoptotic Bodies, ApoBDs, Neuroinflammation, Oxidative Stress, Blood-Brain Barrier, Neuroprotection, Engineered Extracellular Vesicles. Boolean logic operators (AND/OR) were adopted for combined retrieval to accurately screen the target literature focusing on the correlation between EV subtypes and TBI pathological progression as well as their therapeutic translational value.

### 2.3. Literature Inclusion Criteria

Studies that met the following criteria were included in this review: (1) peer-reviewed original research articles and high-quality reviews focusing on the biological characteristics, subtype classification, and cellular origin heterogeneity of EVs in TBI; (2) studies exploring the dual regulatory effects and molecular mechanisms of exosomes, microvesicles, and apoptotic bodies on post-TBI neuroinflammation, oxidative stress, blood–brain barrier damage, and neurodegenerative progression; (3) research on engineered EV modification, targeted delivery systems, subtype-specific isolation technology, and the clinical translational potential of EV-based TBI therapy; (4) studies involving preclinical animal models, cell experiments, and clinical sample verification related to TBI and EVs; (5) English-language studies with complete data, rigorous experimental design, and reliable research conclusions.

### 2.4. Literature Exclusion Criteria

Studies that met any of the following conditions were excluded: (1) letters, conference abstracts, case reports, editorial materials, and reviews with repetitive content and insufficient depth of analysis; (2) studies focusing on other central nervous system diseases (stroke, neurodegenerative diseases, brain tumors) without involving TBI pathological mechanisms; (3) research only discussing general EV biological characteristics without analyzing subtype-specific functional differences and dual regulatory effects; (4) studies with incomplete experimental data, flawed research design, inconsistent conclusions, or low citation frequency and academic value; (5) non-academic gray literature and duplicate published papers.

## 3. Subtypes and Sources of EVs in TBI

### 3.1. Biogenesis of EVs

**Figure 1:**
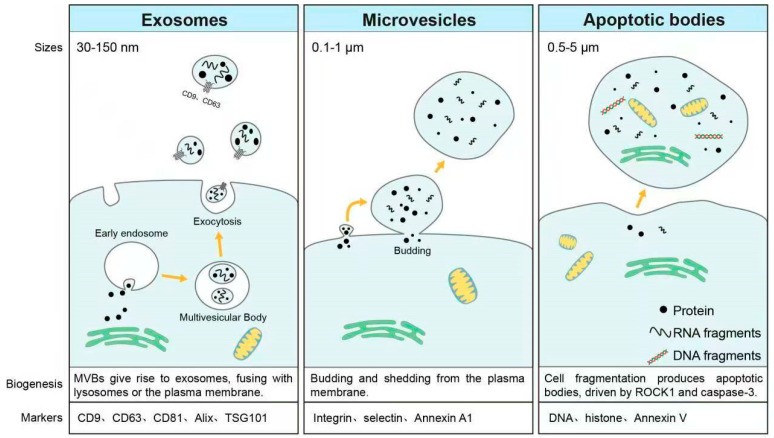
Overview of the dual roles of extracellular vesicle (EV) subtypes in traumatic brain injury (TBI).

According to their biogenesis, formation mechanism, and size, EVs were classified as three major subtypes: exosomes, MVs and ApoBDs; all show different biological functions and roles in both normal and disease conditions [18,19,20] (Figure 1). A core feature distinguishing EV subtypes is their selective cargo packaging and loading mechanisms, which directly dictate intercellular signaling specificity and functional outcomes; the hierarchical cargo sorting processes are tightly regulated by cellular machinery, ensuring targeted delivery of bioactive molecules rather than passive random release.

Exosomes are 30–150 nm in diameter, and they derive from inward budding of late endosomal membranes to form intraluminal vesicles (ILVs) within multivesicular bodies (MVBs). Exosome biogenesis involves the inward budding of endosomal membranes to form intraluminal vesicles (ILVs) within multivesicular bodies (MVBs), followed by MVB fusion with the plasma membrane and release of ILVs as exosomes [21]. Exosomal cargo packaging is a highly regulated process governed by two primary pathways: the endosomal sorting complex required for transport (ESCRT)-dependent pathway and ESCRT-independent pathway. The ESCRT machinery (comprising ESCRT-0, I, II, III complexes and accessory proteins) mediates ubiquitinated protein sorting into ILVs, while lipid raft-dependent mechanisms (enriched in ceramide, cholesterol, and sphingolipids) drive non-ubiquitinated cargo loading, including microRNAs, lipids, and non-coding RNAs. RNA-binding proteins (e.g., hnRNPA2B1, Argonaute 2) specifically recognize and chaperone nucleic acid cargo into MVBs, preventing RNA degradation and enabling targeted loading. These exosomes—which contain tetraspanins such as CD9, CD63, and CD81, as well as proteins, lipids, and RNA—are released and, in turn, modulate recipient cell function; the selective packaging of these cargoes is not stochastic but tailored to the parental cell’s physiological state and stress response.

In contrast, the MVs of 100–1000 nm in diameter are formed by outward budding of the plasma membrane with cytoskeletal remodeling and the gain of membrane asymmetry and exposing the phosphatidylserine (PS) on the surface of the vesicle [22,23]. MV cargo loading occurs concurrently with plasma membrane budding, driven by cytoskeleton rearrangement (actin and myosin contraction) and lipid redistribution. Cytosolic proteins, membrane receptors, adhesion molecules, and nucleic acids are passively and selectively incorporated into MVs based on their subcellular localization and membrane affinity; integrins and surface markers are enriched during budding, enabling targeted docking to recipient cells. MVs are defined by the expression of integrins and other markers allowing them to dock on target cells, and their cargo are composed both of cytoplasmic and membrane components and reflect their cellular origin and functional heterogeneity, with cargo composition directly mirroring the parental cell’s activation status and pathological microenvironment.

ApoBDs, the largest EV subtype at 500 nm to 5 μm in diameter, form during apoptosis, and they include a fragment of the cell with cell debris organelles and genomic material [24]. ApoBD cargo packaging is linked to apoptotic cell disassembly, involving random encapsulation of fragmented organelles, genomic DNA, histones, and apoptotic signaling molecules, with minimal selective sorting compared to exosomes and MVs. Unlike exosomes and MVs that actively participate in intercellular signaling, ApoBDs play a mainly role in clearance of apoptotic cells as they are promptly recognized and phagocytosed in order to prevent release of the potentially toxic intracellular contents into tissues, allowing the preservation of homeostasis [25,26]. Given the distinct biogenesis and functions of EV subtypes, it is essential to recognize their dual roles as both critical regulators of cellular homeostasis and active participants in diverse pathophysiological processes. This duality necessitates their separate analysis when investigating their roles in health and disease.

### 3.2. Cellular Origin Heterogeneity of EVs After TBI

EVs might be released from neuronal, microglial and astrocytic cells or peripheral inflammatory cells following TBI [15,27]. They have unique molecular cargoes depending on the cellular phenotype and pathological reactions following the insult. In terms of heterogeneity, the release of EVs not only represents heterogeneous functions of these cells but also governs cell-to-cell communication in the healing and recovery of TBI [28] (Table 1).

Neuron-derived EVs (NDEVs) contain neuron-specific proteins, including proteins associated with synapses (like synaptophysin); proteins associated with neurodegeneration, including phosphorylated tau [29], the presence of which was used as a marker in one investigation [30]; and other proteins. NDEVs can thus give useful information regarding the degree of cellular injury and neurodegeneration, potentially making them important candidates for injury biomarkers. Neuron-derived extracellular vesicles (NDEVs) also contain multiple microRNAs such as miR-21, miR-212, miR-146, and miR-7, involved in regulating neuroinflammation and neuro injury following TBI [31]. Also, high levels of PrPc, synaptogyrin-3, and Aβ42 (Aβ42) markers in NDEVs are associated with cognitive deficits [32]. These vesicles are not only biomarkers but may also actively participate in neuronal survival and repair in response to stress and therefore demonstrate their dual function of both neurodegeneration and neuroprotection after TBI. MiRNAs, such as miR-21, miR-212 and miR-7a, play a role in the regulation of neuronal injury and inflammation mediated by EV cargo [31].

Concurrently, microglia, the resident innate immune cells of the CNS, are actively mobilized upon TBI and release EVs containing inflammatory molecules, such as interleukin-1β (IL-1β) and complement factors, such as C1q [33]. Microglia EVs (MGEVs) might induce neuroinflammation and thus contribute to neuronal damage, while, depending on their cargo composition, they can also aid in tissue repair and the resolution of inflammation [34]. Interestingly, miR-124-3p in MGEVs has been shown to contribute to regulation of post-traumatic neurodegeneration by targeting the Rela/ApoE signaling pathway and promote the degradation of β-amyloid, thereby produce better cognitive outcomes post-repetitive mild TBI [30]. MiRNAs, including miR-124-3p and miR-711, promote neuronal autophagy and microglia polarization so as to regulate the progression and recovery of TBI [35,36]. These EVs have also exhibited potential to modulate neuroinflammation and enhance functional recovery by promoting hippocampal neurogenesis and attenuating neurodegeneration in experimental models [37]. Moreover, MGEVs enriched with IL-1b, miR-155 and TNF-a have the capacity to stimulate microglia and elicit neuroinflammation capable of generating a systemic immune response and promoting neuroinflammation in the TBI cortex [38].

EVs derived from astrocytes (ADEVs) also complicate this picture as ADEVs consist of metabolic regulatory proteins (e.g., glutamate transporter GLT-1) and molecules implicated in scar formation (e.g., chondroitin sulfate proteoglycans) that may impede axonal regeneration [39]. But ADEVs also deliver neuroprotective molecules, including the heat shock proteins and apolipoprotein E that protect the neurons [40,41]. Recent studies show that miRNA-382-5p in ADEVs regulates astrocyte–neuron communication, resulting in neuronal mitochondrial impairment and apoptosis, which could worsen TBI outcomes [42]. Nonetheless, other molecules, like miR-873a-5p, induce M2 microglial polarization and decrease inflammation; hence, ADEVs also have a possible therapeutic role [43]. In addition, ADEVs with molecules of GJA1-20k and NKILA were reported to exert pro-neuronal effects by diminishing apoptosis and also favoring neuron proliferation [44,45] and inducing the Nrf2/HO-1 signaling cascade in hippocampal neurons, enhancing the levels of antioxidant enzymes and reducing oxidative stress, which results in improved reduced neurobehavioral deficits and neuron survival [46].

Moreover, cerebrospinal fluid-derived EVs (CSF-EVs) and subventricular zone-derived EVs (SVZ-EVs) provide more clues into TBI pathology. CSF-EVs represent the intracellular biochemical milieu after trauma and may act as useful biomarkers for TBI severity and progression [37]. Conversely, SVZ-EVs generated by neural stem cells have been suggested to exert neurogenesis and brain repair-promoting activities by increasing stem cell proliferation and differentiation and are shown to have promising potential for therapeutic recovery post-TBI [38]. Macrophage-derived EVs, specifically LPS-induced EVs, stimulate secondary damage following TBI by upregulating systemic inflammation and CNS/hepatic leukocyte recruitment [47].

**Table 1:** Heterogeneity of extracellular vesicle subtypes: cellular origin, cargo, functions, and TBI outcomes.

Cellular Origin	Cargoes	Functions	Direction of Effects on TBI Prognosis	TBI Outcomes	References
NDEVs	PrPc, synaptogyrin-3, P-T181-tau, P-S396-tau, Aβ42, IL-6	Serve as long-term biomarkers for cognitive impairment, reflecting neuronal damage and dysfunction	Detrimental	Elevated levels in subjects with TBI and cognitive impairment; potential drug targets	[32]
	miR-21, miR-212, miR-146, miR-7a, miR-7b	Mediate neuroinflammation and neuronal injury via EVs; regulate neuronal survival and repair	Context-dependent	Increased neuroinflammation and neuronal injury; altered miRNA expression in brain EVs	[31]
MGEVs	miR-124-3p	Target the Rela/ApoE signaling pathway to regulate neurodegeneration; promote β-amyloid breakdown	Beneficial	Improved cognitive outcomes, reduced neurodegeneration	[30]
	miR-124-3p	Regulate neuronal autophagy by targeting FIP200, preventing excessive autophagy in injured neurons	Beneficial	Improved functional scores, increased neurogenesis	[35]
	IL-1β, miR-155, TNF-α	Activate microglia and initiate neuroinflammation in vivo; propagate systemic immune responses	Detrimental	Exacerbated neuroinflammation, increased secondary injury	[38]
	miR-124	Promote M2 polarization of microglia, inhibit the TLR4 pathway, reduce neuroinflammation	Beneficial	Improved functional recovery, reduced neuroinflammation	[37]
	miR-711	Transfer to neurons to inhibit Itpkb expression, reduce Tau phosphorylation	Beneficial	Improved cognitive function, reduced neurodegeneration	[36]
ADEVs	miRNA-382-5p	Mediate astrocyte–neuron communication, inhibit OPA1 expression, induce mitochondrial dysfunction	Detrimental	Increased neuronal apoptosis, worsened TBI outcomes	[42]
	miR-873a-5p	Promote M2 phenotype transformation in microglia, reduce inflammation via ERK and NF-κB pathways	Beneficial	Improved functional scores, reduced brain injury	[43]
	Unspecified functional protein	Activate the Nrf2/HO-1 signaling pathway in neurons, reduce oxidative stress	Beneficial	Reduced neurobehavioral deficits, increased neuronal survival	[46]
	GJA1-20k	Promote neuronal recovery by reducing apoptosis and enhancing mitochondrial function	Beneficial	Reduced neuronal apoptosis, improved neurologic function	[44]
	NKILA	Transfer NKILA to neurons, promote proliferation, inhibit apoptosis	Beneficial	Reduced brain damage, improved recovery	[45]
Macrophage-derived EVs	LPS-stimulated EVs	Exacerbate systemic inflammation, enhance leukocyte recruitment in the CNS and liver	Detrimental	Increased secondary damage, worsened neuroinflammatory response	[47]

## 4. Neuroprotective Roles of EV Subtypes in TBI

### 4.1. Exosomes

Previously regarded only as inert cellular debris, exosomes have been recently found to be involved in several pathological processes, including inflammation, apoptosis and tissue repair in the CNS. Indeed, these vesicles transport a bioactive molecules like microRNAs (miRNAs), proteins, lipids and nucleic acids, and influence responses following TBI [48,49,50]. Exosomes have been shown to attenuate neuronal damage and facilitate functional recovery following TBI [51]. The anti-neuroinflammatory exosomes’ first priority is to inhibit brain inflammation, which is a characteristic of secondary brain injuries [52]. Microglia, which are active, can both eliminate and exacerbate brain injury after TBIs [53]. Mesenchymal stem-cell-derived exosomes have been reported to stimulate microglial polarization toward the anti-inflammatory M2 phenotype and inhibit proinflammatory cytokine (such as TNF-α and IL-1β) expression [53,54]. Thus, this conversion of the microglia phenotype minimizes inflammatory response and secondary damage to neurons in TBI. BMSC exosomes (bone marrow mesenchymal stem cell-derived exosomes) decrease the expression of pro-apoptotic BAX and pro-inflammatory cytokines TNF-α and IL-1β, meanwhile promoting the expression of the anti-apoptotic protein BCL-2. These exosomes have also been shown to regulate microglial/macrophage polarization, downregulating iNOS and upregulating CD206 and Arg1 to support neuroprotection [55].

In addition to anti-inflammatory effects, exosomes, among many other things, are found to have a key role in supporting neuronal survival and tissue recovery. Exosomes released by astrocytes include antioxidants and proteins (such as S100A10) that act in neuroprotective mechanisms, ranging from protection against oxidative stress to enhancement of BBB integrity [56]. In addition, exosome-mediated transfer of miRNAs (such as miR-21-5p) have been shown to trigger the Akt signaling pathway and reduce neuronal apoptosis after TBI [57]. BMSC exosomes induce neuroprotection, upregulating glutamate transporter-1 and inhibiting the p38 MAPK signaling pathway [58]. hUCMSC (human umbilical cord mesenchymal stem cell) exosomes target various cell death pathways, including apoptosis, pyroptosis and ferroptosis, and induce the PINK1/Parkin pathway-mediated mitophagy after TBI in vitro [59]. hUCMSC exosomes reduce both neuronal death and inflammation in parallel by activating the lncRNA TUBB6/Nrf2 axis after TBI [60].

Exosomes are also responsible for restoring BBB integrity after TBI [61]. Exosomes originating from both astrocytes and MSCs may help to stabilize the BBB, since proteins and lipids derived from these vesicles can help maintain endothelial cell junction integrity [62], which in turn helps to restore BBB integrity, preventing additional injuries to neuronal structure as well as reducing edema leading to better functional recovery post-TBI. Exosomes participate in inducing neurogenesis and tissue healing, thereby promoting regenerating of TBI brains. As an example, BMSC exosomes can promote neurogenesis in the hippocampus and promote angiogenesis in the peri-traumatic area [63].

This therapeutic potential for the exosomes is further evidenced by their capability to cross the BBB to deliver therapeutic cargo to the injured brain region. In addition, the exosome-mediated delivery of miRNAs promotes autophagy, restores mitochondrial function, and reduces oxidative stress management after TBI. For instance, exosomes loaded with miR-124-3p have been demonstrated to inhibit neuronal apoptosis by targeting pro-apoptotic genes and activating survival pathways, thus promoting long-term neurological outcome after TBI [64]. Exosomes can activate Nrf2/HO-1 signaling, reducing oxidative stress and neuronal apoptosis [46].

### 4.2. Microvesicles

Compared with exosomes, MVs are less uniform and comprise a wider range of vesicles that participate in TBI pathophysiology. Indeed, there is a growing body of literature indicating that different cell origin-derived MVs, such as platelets and endothelial and neuron-derived MVs, contribute to an integral regulation of immune response and regulation of vascular homeostasis in TBI [65,66].

The main functions of MVs in TBI include immune modulation and coagulation abnormality. MVs can modulate platelet activity, which is an important function of hemostasis reaction after TBI. After TBI, MVs induce activation of the ADP receptor of platelets (ADP receptor P2Y12), resulting in platelet dysfunction (impaired aggregation) [67]. This type of MV-induced change in platelet activity has been shown to be involved in the coagulopathy typically found in TBI patients, acting synergistically on the increased possibility of intracranial hemorrhage [67,68]. In addition, MVs freely circulating through the body often have procoagulant molecules bound to them, compounding this effect and contributing to treating bleeding issues in TBI [69]. Experimental clearance interventions for these MVs have shown some promise for decreasing coagulopathy and enhancing the balance of hemostasis thus have therapeutic relevance. New preclinical data indicate that a one-time dose of lactadherin administered either 30 min prior to or 30 min after TBI can enhance phosphatidylserine-mediated clearance of circulating MVs, thus preventing coagulopathy and improving neurological outcomes [70]. In addition to the coagulation, MVs released from brain endothelial cells play a fundamental role in the disruption of the BBB subsequent to TBI. Endothelial-originating MVs carrying the tight junction proteins, e.g., occludin and intercellular adhesion molecule-1 (ICAM-1), are released into extracellular space that is associated with BBB integrity [71]. Furthermore, endothelial-derived MVs act not only as biomarker of the BBB injury but also directly contribute to vascular remodeling and inflammation.

Further, neuroinflammatory reaction after TBI is significantly affected by MVs derived from neurons and glial cells. NDMVs loaded with complement components, such as C1q, can trigger microglial activation and recruit peripheral immune cells at the site of injury, exaggerating inflammation and facilitating neurodegeneration [33]. MVs loaded with cytokines, proteins, miRNAs, etc., modulate the immune response and vascular permeability. For instance, von Willebrand factor (VWF)-bound MVs increase vascular permeability and coagulopathy, whereas apoptotic-cell-derived MVs contribute to cell debris and inflammation clearance via the phagocytic process [72]. This positive–negative MV-induced functional dichotomy reveals the dual functions that MVs may play in the pathophysiology of TBI and the future potential for targeted therapies with certain MV populations to have a positive impact on cerebral damage.

### 4.3. Apoptotic Bodies

ApoBDs, which mark the end result of apoptotic cell death, are implicated in the response of cells after TBI. Apoptosis is a critical mechanism for disposal of defective or unnecessary cells through a regulated process that avoids triggering inflammatory responses [73,74]. After TBI, the apoptotic program is activated in neurons and glial cells, resulting in formation of ApoBDs. These extracellular particles encapsulate cellular debris, such as cell damaged and released fragments of DNA and the intracellular organelles, and are recognized by phagocytic cells, such as macrophages and microglia, given the externalization of the phosphatidylserine on their cell surface [75,76]. The efficient elimination of ApoBDs is fundamental for the maintenance of the tissue homeostasis and preventing the release of pro-inflammatory intracellular contents that could provoke secondary injury.

After TBI, ApoBD formation is a typical feature of neuronal apoptosis that contributes to secondary brain damage and delayed neuronal cell loss [77]. Caspase activity, especially caspase-3, is critical to the damage to the neuronal nucleus and the subsequent ApoBD production. The occurrence of ApoBDs in the cortex and hippocampus have been reported using TUNEL staining and caspase-3 immunoreactivity as an indicator of ongoing neuronal injury [78]. Caspase-3 is activated after cleavage from the upstream initiator caspases, caspase-8 or casapse-9, and mediates a series of events responsible for the morphological changes seen in apoptosis [79,80]. These hallmarks (cell shrinkage, chromatin condensation, membrane blebbing, and ApoBD formation) and a similar observed order (first, the cell shrinkage; second, chromatin condensation; third, ApoBD formation; and fourth, membrane blebbing) are in keeping with cell damage observed to date, which is associated with the proteolysis activation of the cell-death program that mostly occurs during the early post-TBI phase (days 1–3).

ApoBDs modulate neuroinflammation as fast clearance by microglia impedes the neuroinflammation promoted by the accumulated cellular debris and release of pro-inflammatory cytokines; on the other hand, slow or defective clearance of ApoBDs instigates sustained neuroinflammation, which aggravates secondary brain injury and deteriorates clinical outcome [81]. Thus the efficient phagocytic process can contribute to the attenuation of TBI damage. Although the function of ApoBDs is clearly established in TBI, the molecular mechanisms regulating ApoBD formation, their signalization and degradation remain subjects of intense investigation.

In the context of TBI, ApoBDs have been primarily characterized as morphological end-products of apoptosis and as substrates for phagocytic clearance. Their formation, as evidenced by TUNEL staining and caspase-3 activation, serves as a reliable indicator of ongoing neuronal and glial cell death in both experimental models and post-mortem human tissue [78,79]. Whether ApoBDs also act as active mediators of intercellular communication after TBI, however, remains an open question. In other pathological contexts, ApoBDs have been shown to transfer bioactive molecules—including DNA, RNAs, and proteins—to recipient cells and to modulate immune responses [27,80]. Future investigations employing subtype-specific isolation and functional assays will be necessary to determine whether ApoBDs in the TBI brain serve as bona fide intercellular messengers or remain confined to their established role in apoptotic cell clearance. Overall, origins, subtypes, and functions of extracellular vesicles are shown in Table 2.

## 5. Negative Impacts of EV Subtypes in Neurodegenerative Pathologies Following TBI

### 5.1. Cross-Cellular Propagation of Pathological Proteins via EVs

TBI also commences dissemination of toxic proteins like tau and amyloid beta (Aβ), spreading neurodegeneration and contributing to long-term effects [82,83,84]. EVs have been recognized as mediators in propagating the toxicity of proteins by facilitating cell-to-cell and BBB crossing [85,86]. Emerging evidence indicates that EVs loaded with tau and Aβ can spread pathologies between different brain areas further, deepening the consequences of TBI and leading to chronic neurodegenerative diseases (such as chronic traumatic encephalopathy and Alzheimer’s disease) [87].

After TBI insults, tau-containing EVs are released by neurons and promote tau pathology in healthy neighboring neurons by facilitating the dissemination of neurofibrillary tangles, a hallmark of tauopathies [86,88,89,90,91]. This mechanism of EV-mediated tau dissemination has been demonstrated both in animal models and clinical studies, and elevated tau levels in EVs in TBI patients are associated with poorer cognitive outcomes [92,93]. This dissemination of tau pathology indicates that EVs are likely key vehicles in the progression of tau-mediated neurodegenerative diseases and portends therapeutically relevant use of targeting EV secretion as a method to arrest or attenuate disease.

Likewise, Aβ, a protein that is closely linked to other neurodegenerative diseases, was also demonstrated to be transported through EVs upon TBI. In particular, soluble Aβ (Aβ1-42) is secreted from neurons and astrocytes as a response to brain injury, and EVs containing Aβ1-42 amplify neuronal damage by inducing synaptic loss and mitochondrial dysfunction [94]. Furthermore, the deposition of Aβ in microglia-derived EVs is believed to be a reaction of the brain to clean up this toxic substance, whereas its emission may also further spread neurodegeneration [95]. The interaction between microglia and Aβ containing EVs is part of the reaction cascade of the brain toward injury, and the protective responses may also add to disease progression. Finally, EVs’ potential to cross the BBB contributes to their involvement in systemic propagation of pathological proteins as evidence suggests that brain-derived EVs containing tau and Aβ can be found in peripheral circulation after TBI [96]. This leads us to hypothesize that EVs are involved in system wide neuroinflammation and coagulopathy, playing a role not only as diagnostic markers of active neurodegeneration but also as a prognostic factor of post-TBI-related diseases, i.e., chronic neuroinflammation and cognitive impairment.

### 5.2. Amplification of Inflammatory Cascades in TBI Through EVs

TBI also generates a neuroinflammatory response that in the case of long-term persistence could induce secondary brain damage and neurodegenerative diseases. Post-TBI, microglia can switch to a pro-inflammatory M1 phenotype to secrete cytokines (e.g., IL-1β, TNF-α, and IL-6), ROS and nitric oxide and products that worsen inflammation locally and recruit immune cells from the peripheral system [12]. In addition, reactive astrocytes release cytokines such as IL-1β, IL-6, and CCL2 (MCP-1) to activate the NF-κB pathway and upregulate other inflammatory mediators [97,98]. Both microglia and astrocytes release exosomes (small vesicles carrying extracellular cytokines such as pro-inflammatory proteins or miRNAs, namely, miR-155, miR-30d and so on) to transmit the inflammation cascade and initiate a positive feedback loop between pro-inflammatory state in astrocytes and microglia [99,100].

After breaking through the BBB, EVs containing proinflammatory cytokines and miRNAs leak out and move to peripheral organs via peripheral circulation, further inducing systemic inflammation and organ failure [16,101]. EVs fuse to target cells, entering their target cells via endocytosis to activate the NF-κB and MAPK signaling pathways and downstream signal pathways [102]. Among these exoRNAs are pro-inflammatory miRNAs, miR-21-5p and miR-155, which modulate the expression of genes in the target cells, favoring inflammation and preventing tissue repair [103]. For instance,. miR-21-5p-loaded EVs induce M1 polarization of microglia, which favors cytokine secretion, while suppressing neuronal neurogenesis.

We observed that the inflammasome was upregulated in the cerebrospinal fluid of patients with severe TBI [104]. The NLRP3 inflammasome is induced by damaged cells’ danger signals and activation of caspase-1 as well as the release of cytokines such as IL-1β and IL-18 [105,106]. These cytokines promote pyroptosis and release of inflammatory mediators that further trigger further inflammatory responses. It has been demonstrated that microglia and other glia EVs also have NLRP3 inflammasome components; these are hypothesized to activate the inflammasome, thereby linking EV mediated signaling to long-lasting tissue damage following TBI [102].

MiR-21 is upregulated in the neurons and EVs surrounding the injury site, indicating that it may be released from neurons as cargo in EVs following TBI [31]. This finding uncovered a new mechanism of cell–cell communication in TBI, in which neurons can release EVs with miR-21 to regulate the inflammatory response in cells surrounding the neurons. Knowing of these mechanisms of EV-mediated neuroinflammation will not only underscore the intricacy of the post-TBI inflammatory cascade but will also offer new therapeutic targets for neuroinflammation reduction and recovery facilitation.

### 5.3. Oxidative Stress in TBI and the Role of EVs

ROS production and deficits of the antioxidant mechanisms in cells after TBI produce oxidative stress, which is a significant source of secondary injury of neurons [5,107]. After a TBI, due to mitochondrial dysfunction, excessive production of ROS overwhelms the brain antioxidant systems and induces multiple oxidative injuries [108]. These lead to modification of the cellular lipids, proteins and DNA, all leading to neuronal death and blood–brain barrier (BBB) breakdown as well as an increase in general severity of the injury [98].

Mitochondrial dysfunction can be considered the hallmark of TBI-induced oxidative stress. Following TBI, axonal injury and neuronal membrane lysis allow the entry of calcium ions into neurons, thereby activating different signaling molecules, resulting in upregulation of ROS production [109]. This subsequent increase in ROS further triggers lipid peroxidation, which means that ROS can react with cellular membranes, resulting in the production of harmful byproducts: F2-isoprostanes and reactive carbonyl species [110,111]. These metabolites also further propagate oxidant damage by altering cell structures, inducing inflammation, causing apoptosis and worsening neuronal injuries and dysfunction. Oxidative stress as well as secondaries like excitotoxicity, neuroinflammation, and apoptosis contribute to further worsening of long-term TBI outcomes [112].

EVs contain cargoes like proteins, lipids, miRNAs and mitochondrial DNA that can be transferred from one cell to another, induce intercellular communication, and drive propagation of pathological processes following TBI [107,113]. Oxidative stress-wise, EVs are meaningful mediators that transfer the oxidative damage signal from one cell to another, thereby propagating the trauma. Recent reports noted that EVs harvested from neurons and glia after TBI contain a large quantity of oxidized molecules—oxidized proteins and lipids—that are known to exert direct pro-oxidative stress on recipient cells [100]. MtDNA released into the extracellular space as a consequence of mitochondrial damage can act as key biomarker for mitochondrially induced oxidative stress. It has been demonstrated that mtDNA bound to EVs is present in increased concentrations in both CSF and serum after TBI and can serve as a biomarker of mitochondrial damage [110,114]. EV-associated mtDNA uptake by adjacent cells may result in additional mitochondrial damage and increase oxidative stress, further propagating neuronal dysfunction and neurodegeneration. The ability of EVs to transport and deliver mtDNA across the BBB further supports the implications of the role of EVs in the systemic inflammation and oxidative injury after TBI.

Apart from direct propagation of oxidative damage by EVs, the influence of EVs on the antioxidant response in the brain has also been described. EVs from neural stem cells (NSCs), astrocytes and microglia contain antioxidants like superoxide dismutase (SOD) and catalase that can be imported to the neighboring cells as a means of neutralizing oxidative stress [98]. For instance, astrocyte-derived EVs are reported to pro-activate the Nrf2/HO-1 signaling pathway within neurons (this signaling pathway also has pivotal contributions to the control of the cellular antioxidant defense system and is involved in neuronal protection against oxidative insults [46,100]) and may be transferred as antioxidant EVs, representing a treatment that possibly lessens the oxidative stress and enhances neuronal survival following TBI [115]. Nevertheless the therapeutical role of such vesicles is complex since they may carry molecules with proinflammatory and pro-apoptotic properties related to advancement of neurodegeneration.

### 5.4. Key Determinants of the Dual Roles of EVs in TBI

The functional dichotomy of EVs in TBI—spanning neuroprotection and exacerbation of injury—is governed by an interplay of multiple interdependent factors rather than by a single intrinsic property of the vesicles. Six major determinants can be delineated based on current evidence. First, the cellular origin imprints EVs with distinct molecular signatures: neuron-derived EVs may simultaneously carry neuroprotective miRNAs (e.g., miR-124-3p) and pathological proteins such as phosphorylated tau, while microglia-derived EVs can shift between pro-inflammatory (IL-1β and miR-155) and pro-resolving (miR-124-3p and miR-711) cargoes depending on the activation state of the parent cell [31,32,39]. Second, the EV subtype dictates the mode of biogenesis and the range of cargo that can be packaged; exosomes preferentially shuttle miRNAs and signaling proteins, microvesicles are enriched in plasma membrane receptors and procoagulant molecules, and apoptotic bodies primarily contain fragmented organelles and genomic material, thereby determining their predominant functional roles in intercellular communication versus clearance [19,21,73]. Third, molecular cargo composition is a direct effector of outcome: the same EV subtype can produce opposing effects depending on whether it delivers anti-apoptotic miRNAs (e.g., miR-21-5p activating Akt), antioxidant enzymes, or, conversely, aggregated tau and Aβ species that propagate neurodegeneration [58,87,91]. Fourth, the post-injury time phase critically influences the prevailing EV population; acute-phase EVs are often enriched in damage-associated molecular patterns that amplify inflammation and coagulopathy, whereas subacute and chronic phases see an emergence of EVs carrying pro-resolving and regenerative factors [55,64,103]. Fifth, recipient cell type adds another layer of complexity: for instance, miR-21-5p delivered by exosomes can exert anti-apoptotic effects in neurons while simultaneously polarizing microglia toward a pro-inflammatory M1 phenotype [58,104]. Sixth, the surrounding inflammatory microenvironment—including the local cytokine milieu (e.g., IL-4 and IL-13 versus IL-1β and TNF-α) and the activation status of both donor and recipient cells—shapes the functional consequences of EV transfer, determining whether microglial EVs, for example, promote M2 polarization and tissue repair or M1-driven neuroinflammation [35,44,101]. Collectively, these six determinants underscore that the net effect of EVs in the injured brain is highly context-dependent and cannot be reduced to a single molecular or cellular parameter. Recognizing this complexity is essential for the design of EV-based diagnostic and therapeutic strategies, as well as for the accurate interpretation of experimental data in the TBI field.

## 6. Therapeutic Potential of Engineered EV Subtypes

### 6.1. Targeted Modification Strategies for Engineered EVs

Because of the natural functions of EVs in intercellular communication and the ability of EVs to convey bioactive molecules, such as proteins, lipids, RNA, mitochondria or even viruses, natural EVs have been proposed as promising targeted drug delivery systems. However, the heterogeneity between EV populations, the difficulty of routinely producing therapeutic stable EVs, and the complexity per se of their contents limit the clinical utilization of natural EVs [28]. Artificial EVs are even emerging as potential tools for precision medicine, such as oncology, neurodegenerative conditions, and regenerative medicine [116,117]. They may be sourced naturally from almost any cells, and their surfaces can accommodate a wide range of molecules, including RNAs, proteins, and lipids. An important limitation of applications of EVs to therapy is specificity to the target tissue [118]. A high degree of specificity allows for lower doses with fewer off-target effects and better therapeutic success. Several strategies to augment targeting ability of EVs through either altering the vesicles themselves or altering their sources (the parent cells from which they are obtained) have been devised. Such strategies were able to generate the possibilities for an optimized, target-oriented therapy for diseases.

Endogenous genetic modification of donor cells improves the characteristics of EVs produced by these cells. Through transfection of cells with plasmids or viral vectors with the expression of specific proteins or peptides, the surfaces of the EVs can be directed to confer characteristics that enhance the EVs’ targeting ability [119]. For instance, the Lamp2b protein, a naturally occurring exosomal component, can be conjugated to targeting peptides such as rabies virus glycoprotein for delivery into the brain, targeting brain cells for treatment of neurodegenerative diseases such as Alzheimer’s disease [120], but gene transfection is susceptible to such obstacles as low transfection efficiency and genetic instability in transfectants. Gene expression variability between donor cells can drive the heterogeneity of the yielded EVs, which can contribute to unpredictability in therapeutic activity. Yet all these disadvantages have not prevented the application of endogenous strategies, which remain an attractive option because they are easier and simpler than exogenous approaches, relying as they do on natural biogenesis pathways that confer stability.

Exogenous approaches directly manipulate EVs once they are harvested from donor cells. They utilize physical methods, e.g., electroporation, sonication, extrusion, and freezing–thawing, to produce transient pores in the EV membrane so as to load them with therapeutic cargo, e.g., RNA, small molecules, or proteins [116]. For example, electroporation, where a transient pore is created in the EV membrane by an electric field, allowing for molecules to diffuse across the EV membrane, is frequently employed. Electroporation has been used effectively to load RNA and small molecules into EVs for delivery [121,122]. However, electroporation may compromise EV membrane integrity, thereby reducing therapeutic efficacy of the vesicles, and can affect the therapeutic application. Sonication takes advantage of high-frequency sound waves to produce shear forces that can load hydrophobic drugs into EVs by bridging hydrophobic molecules with the lipid bilayer of the vesicle [123]. Nonetheless, such an approach can lead to irreversible damage in EVs, impeding its common use. Extrusion and freeze–thaw cycles are also exogenous modification approaches based on the application of physic stress to induce changes on EV membranes and then promoting cargo loading [124,125]. Extrusion relies on the expulsion of EVs through a small pore, disrupting the structure of the membrane, which facilitates encapsulation of the cargo. Although extrusion has demonstrated a better loading capacity than the rest of the methods, multiple applications may result in damage to the vesicle membrane and EV aggregation. Overall, although these approaches are limited, they represent an interesting strategy for improving EVs’ therapeutic capacity.

Surface engineering refers to decorating targeting peptides, antibodies, or ligands on the surfaces of EVs, which can facilitate interactions with the receptors of specific target cells and therefore enhance the targeting specificity of engineered EVs [126,127]. For example, peptides such as targeting low-density lipoprotein receptor-related protein 1 (Angiopep-2) have been loaded into EVs to promote these EVs to transit the BBB and target brain tissue [128,129]. In an Alzheimer’s disease animal model, engineered autologous dendritic-cell-derived exosomes with LAMP-2b and RVG expressed on their surface expressed systemically downregulated β-secretase-1 expression in neurons, microglia and oligodendrocytes [120]. Moreover, miR-124 was loaded onto RVG expressing exosomes by electroporation, which delivered miR-124 to the infarct zone effectively and enacted cortical neurogenesis in an ischemic injury mouse model [130]. This modification has been employed to manufacture exosome-based drug delivery systems for the treatment of Alzheimer’s and Parkinson’s diseases as neurodegenerative disorders. Surface modification can also be realized via chemical conjugation approaches (e.g., click chemistry), enabling targeting ligands to be covalently tethered to EV surfaces.

### 6.2. Subtype-Specific Clearance Techniques for EVs

Considering the heterogeneity of EV populations, characterization of EVs of interest and specific subgroups of EVs is important for biomarker search and target therapy [131]. Owing to their nanoscale characteristics, EVs are present at high concentrations (10^10^–10^12^ particles/mL) in clinical samples, which poses challenges to purification and recovery [132]. To overcome the challenges of isolating specific EV subpopulations, novel methods for their selective isolation were developed. A promising technique for isolation is immunomagnetic nanopore sorting, which gives a high throughput and efficient way to sort target EVs according to their specific surface markers [133]. Immune magnetic nanopore sorting by trajectory-etched magnetic nanopore (TENPO) chips is a high-throughput protocol for effective separation of target EVs by their specific surface markers [134,135]. The concept of this approach is to label EVs with a biotinylated antibody directed against target-surface marker in advance and conjugate antibody labeled EVs to MNPs, forming a complex, which can then be divided based on the magnetophoretic force exerted by them when passing through magnetic nanopores; EVs highly labeled against the target marker can be clearly captured and separated from the bulk [132]. The method is more than an order of magnitude less susceptible to background contamination than other existing approaches and maintains the throughput necessary to recover EV target subpopulations at a high percentage.

Immune magnetic nanopore sorting technology can be applied to recover the specific EV subtypes in which disease biomarkers are over-enriched (e.g., lung cancer-related EVs, pancreatic cancer-related EVs and liver cancer-related EVs) as a useful approach in early disease diagnosis or evaluation of treatment efficiency. And this technology is easily modifiable by adjusting pore sizes, membrane quantity and flow rate to facilitate the recovery of EVs while being minimally affected by unwanted EV subtypes [136]. Furthermore, to further increase the specificity and yield of EV isolation, immunomagnetic sorting is frequently accompanied by various other separation procedures such as size-exclusion chromatography, ultracentrifugation, or affinity-based capture. For example, immuno-affinity isolation by use of anti-bodies or aptamers to capture surface proteins present on EVs has been extensively used to select EVs based on the presence of surface markers, including CD9, CD63 and tetraspanins [137]. These methods can add a further level of specificity when paired with immunomagnetic sorting, such that only EVs of interest are caught, reducing non-specific binding and co-isolation of spurious particles. These approaches have started to become more personalized to the different applications in the clinic. As another illustration, to isolate cancer-derived EVs one can use antibodies against tumor-specific antigens to capture vesicles that transport biomarkers indicative of metastatic processes [138] or antibodies against neuronal or glial cell markers to isolate subpopulations of EVs that are implicated in neuroinflammation and/or in synaptic dysfunction [139] for neurodegenerative diseases.

## 7. Challenges and Future Directions

### 7.1. Clinical Translation Barriers for EVs

EVs can transport proteins, lipids and nucleic acids to target cells in a direct manner, hence representing an elegant alternative to conventional cell therapy and offering encouraging potential in disease treatment and tissue repair. EV biology complexity and production, standardization and regulation hurdles have slowed down the progress toward EV-based therapeutics and impose significant challenges in translational EV therapeutics toward clinics [140,141]. Though EVs have been postulated to be involved in cell signaling, immunomodulation, and tissue regeneration activities, little is known about the mechanisms through which they exert their specific efficacy.

As researchers have less frequently explored how they gain their physiological functions, it is essential to monitor and detect EVs in in vivo environments. Radioactive labeling, which is the current tracking strategy for EVs, can change the original status of EVs, making it difficult to analyze their in vivo behavior [142]. Though promising, fluorescence resonance energy transfer (FRET) and other microscopy methods are limited by their technical capabilities, specifically their poor sensitivity and resolution, thereby precluding full characterization of EV interactions within the more complicated biology [143].

Due to the variance of EVs (in regard to size and surface markers), standardized manufacturing processes with reproducible results are still challenging to establish. While methods like ultracentrifugation are feasible and widely used in research settings, they pose poor efficiency along with a poorly defined number of isolated EVs since it may differ considerably among batches [144], hindering their application to the clinical setting due to the lack of batch-to-batch reproducibility. Although newer techniques like tangential flow filtration provide increased reproducibility and purity, they are still plagued by factors such as lipoprotein contamination [145].

Thus, characterization techniques for EVs such as electron microscopy, mass spectroscopy, and nanoparticle tracking analysis contribute significantly to verifying the purity and quality of EV preparations [146,147]. Nevertheless, such techniques are labor-intensive, time-consuming, and sometimes not capable of providing sufficient details to satisfy regulatory requirements for clinical applications. A lack of standardized protocols for EV characterization is a limiting factor when comparing one study with another and thus makes it difficult to reliably compare, reproduce and verify the results obtained with EV therapies. As EVs are among the most complex biological entities, any storage procedure can adversely influence the physical–chemical properties and subsequently functional activities [148,149]. For example, ultra-low-temperature freezing has been demonstrated to induce alterations in size distribution as well as decreased particle concentration over time, especially when repeatedly executing freeze–thaw cycles [150]. More recently, lyophilization (freeze-drying) has been investigated as a solution for long-term storage and shipping of EV-based therapeutics with encouraging outcomes with respect to stability and activity of their cargo [151]. Nevertheless, there is still more work to do regarding the potential effects of lyophilization on EV membrane integrity and thus therapy.

Though EVs from MSCs have made significant progress in preclinical and clinical investigations because of their immunomodulatory and regenerative nature, they have also raised some concerns because those EVs from cancer cells may carry cancerous cargo and may fuel tumor growth. In addition, the presence of heterogeneous EV content poses a threat as foreign unwanted bioactive molecules can have undesirable effects as well as invoke a specific immune response [152,153]. Moreover, optimal dosages and administration pathways of EV-based treatments remain largely unknown [154]. It is challenging to define therapeutic EV doses as they are prone to variation in EV load and bioactivity depending on EV origin and the method of isolation. Potential risk assessment for off-target effects, immunogenicity and long-term toxicity require extensive preclinical investigation. Important potential safety concerns for EV-based therapies will necessitate rigorous safety assays at regulatory bodies such as the FDA, including testing for purity, contamination and bioactivity. Achieving regulatory compliance is essential for translational implementation of EV-based therapeutic applications in the clinic.

### 7.2. Emerging Interdisciplinary Fields in EV Research

The joint application between transcriptomics, machine learning, and artificial intelligence fields has broadened the perspective of EV studies regarding therapy and diagnostics. Analysis of EV RNA cargo compositions on the single-particle level (10X genomics-based) reported inherent heterogeneity of the EV RNA cargo profile and determined gene expression variations between EV subtypes from K562 cells and MSCs [155]. Surprisingly, proteins encoded by ribosomal and mitochondrial genes were predominant in EVs, while the presence of different cell-type-specific markers provided a marker of heterogeneity of the pool of vesicles.

ML algorithms that can operate over many large datasets are increasingly used to classify and predict EV subtypes, to identify cellular origins, and to discern role in disease. For example, ML has been coupled with surface-enhanced Raman scattering and electrochemical techniques to improve detection and characterization of tiny EVs [156]. Moreover, AI-driven technologies (such as supervised/semi-supervised/unsupervised learning models) are changing the paradigm of creation of the drug delivery systems by maximizing EV-based therapeutics. For instance, supervised learning permits prediction of drug response using EV-related training models that can further optimize the drug delivery systems for particular therapeutic targets [157,158]. On the other hand, the use of unsupervised learning strategies is indispensable in discovering new EV cargo molecules, revealing new mechanisms of EV uptake, and facilitating the design of more efficient drug delivery platforms. The synergistic application of AI models with high-throughput screening methods assists the mining of large datasets for rapidly discovering potential drug candidates and improving the accuracy and efficiency of EV-based therapy [154]. Unsupervised machine learning has also been explored in recent data, showing large correlations between certain types of EV surface antigens and CVRF for use in immuno-profiling intravascular EVs, adding more weight to EV profiling as a therapeutic stratification tool in disease state and risk estimation [157].

Further, the combination of EV research/medical research with AI/ML that we touched upon above reaches beyond drug delivery applications to cancer therapy and diagnostic biomarker discovery [157,158]. Cancer-cell-derived EVs receive more attention due to their association with tumor propagation, metastasis and immune control. Both AI and ML are being used to optimize approaches to enriching and profiling these vesicles, with an increased understanding of the role they play in oncogenesis and metastasis. AI-enabled enrichment approaches along with EV cargoes can be used to enhance diagnostics of cancer, discover new targets of therapy and personalize therapy in the setting of precision medicine [154,157,158]. This bioinformatics crossover effort is pertinent, on the one hand, to expanding our knowledge of EV biology and, on the other hand, can reveal the impact the joint usage of artificial intelligence/machine learning can have in other aspects of biomedical science and clinical practice in general. The therapeutic potential and translational barriers of engineered extracellular vesicles are summarized in Table 3.

## 8. Conclusions

The positive/negative regulation of subtypes of EVs for TBI further highlights the complicated pathophysiology of TBI. EVs, including exosomes, MVs and ApoBDs, possess positive and negative activities, and whether they are beneficial or harmful to TBI depends on cellular origins, protein cargoes, and the fluctuating microenvironment. EV subtypes operate at the level of the secondary biological response to TBI, not on the initial mechanical insult. For example, exosomes can secrete miR-21-5p, which activates the Akt signaling pathway and suppress neuronal apoptosis while at the same time propagating pathologic proteins such as tau and thereby accelerating neurodegeneration [54]. MVs can carry antioxidants to protect neurons [55], but they can also act by activating platelets and breaching the blood–brain barrier through procoagulant molecules [56,57]. The contrasting roles mentioned above clearly show that such classification by only surface markers (e.g., CD63/CD81) in standard exosome study assays cannot reflect the whole function repertoires of different EV subpopulations. Recently, it was demonstrated that astrocyte MVs can include neuroprotective miRNAs like in an exosome, and the pro- or anti-inflammation role of the microglial EVs is regulated by the cytokines in the surrounding environment, like IL-4 or IL-13.

Passive clearance to active regulation of EVs in TBI thus requires further progress in technology of varied fields such as engineering to further improve the delivery to the brain of exosomes with RVG-LAMP2b targeting manipulations and designing selective nanotraps by DNA origami to capture pathological subtypes such as that harboring phosphorylated tau, but clinical translation of such efforts is limited due to interspecies differences. For instance, for mouse origin EVs, a higher drug-loading efficiency is observed compared with that from human origin EVs, and since there might exist species-specific differences in the penetration of the drug from human brain cells to the blood–brain barrier, it is essential to perform preclinical validation using humanized brain organoids or transgenic models.

Beyond technological innovation and preclinical optimization, rigorous standardization and regulatory compliance are critical for translating EV-based TBI therapies to the clinic, requiring targeted advances in GMP implementation, unified protocols, and regulatory alignment. For TBI-focused clinical-grade EV manufacturing, core GMP standards must be enforced: rigorous donor/cell line qualification, serum-free defined media, closed bioreactor systems for scalable production, and real-time quality assurance to minimize batch variability and contamination risks. To ensure cross-laboratory reproducibility, standardized isolation and characterization workflows are mandatory: adopting GMP-compatible purification (e.g., tangential flow filtration coupled with size exclusion chromatography) and a core panel of assays (NTA size analysis, marker Western blot, and sterility testing) instead of overreliance on single physical metrics. Regulatory pathways, particularly FDA guidelines, are pivotal to de-risking clinical translation. The FDA classifies EV therapies as 351 biological products, requiring robust preclinical/clinical data on safety, purity and efficacy, with no approved exosome therapies for human use to date. Developers must follow FDA EV draft guidance, complete thorough pre-IND studies (immunogenicity, biodistribution, and neurotoxicity), and align with ISEV/ME-HaD SOPs to streamline regulatory review and unify global standards.

High-sensitivity in vivo tracking technologies, including near-infrared-II fluorescent probes to sub-types of EVs, must be developed to resolve spatiotemporal distribution and the functional switching mechanisms of EVs. Also, a guideline in GMP-compliant production relying on functional activities such as neurite outgrowth promoting ratio instead of physical parameters (such as the size of particles) must be established. Interdisciplinary work will be key to the effort. Single-cell EV sequencing can expose the functional heterogeneity of the intra-subtype level, and the application of AI models may be used to forecast a correlation between their cargo and function, facilitating precise isolation of the subtype. Only after the integration of technology innovations and standardization will EV subtype research shift from mechanistic discovery to the patient bedside to lay the foundation of personalized and precise therapeutics for TBI.

Finally, it is important to acknowledge that a substantial portion of the mechanistic understanding summarized in this review originates from preclinical models, and the direct evidence linking specific EV subtypes and cargoes to functional outcomes in human TBI patients remains limited. Extrapolations from stroke, Alzheimer’s disease, and other neurological conditions provide valuable insights but must be validated in TBI-specific clinical cohorts. Addressing the current gaps in clinical evidence, standardizing EV isolation and characterization methodologies, and developing human-relevant model systems are essential next steps for translating the dual roles of EV subtypes into clinically actionable strategies for TBI.

## Figures and Tables

**Table 2:** Origins, subtypes, and functions of extracellular vesicles in TBI.

EV Subtype	EV Origins	EV Function	Direction of Effects on TBI Prognosis	Notes	References
Exosomes	BMSCs	Modulating microglia/macrophage polarization	Beneficial	Polarization regulation effect varies with MSC culture conditions and exosome purification protocols	[55]
	BMSCs	Attenuating neurological injury and improving functional recovery	Beneficial	Dose-dependent curative effect confirmed in pig and rat TBI models	[51]
	MSCs	Reducing neuronal loss, neuroinflammation, and enhancing angiogenesis/neurogenesis	Beneficial	Repair potency differs across different mesenchymal stem cell sources	[63]
	Astrocytes	Protecting against oxidative stress and apoptosis via Nrf2 signaling activation	Beneficial	Antioxidative capacity is related to the activation degree of donor astrocytes	[46]
	BMSCs	Promoting anti-inflammatory microglial polarization	Beneficial	Efficacy fluctuates with acute/chronic TBI pathological stage	[82]
	BMSCs	Neuroprotection via increased GLT-1 in astrocytes, blocking p38 MAPK pathway	Beneficial	Verified in preclinical rodent TBI experiments	[58]
	HucMSCs	Reducing neuron death, apoptosis, pyroptosis, and activating PINK1/Parkin mitophagy after TBI	Beneficial	Protective effect varies at different oxidative stress levels	[59]
	HucMSCs	Neuroprotection by inhibiting inflammation and ferroptosis via TUBB6/Nrf2 pathway	Beneficial	Functional data mainly from in vitro neuronal injury model	[60]
Microvesicles	/	VWF-bound MVs enhance vascular leakage and coagulopathy	Detrimental	Vascular injury aggravation is closely associated with BBB damage severity	[72]
	/	Lactadherin enhances phagocytosis-mediated microvesicle clearance	Beneficial	Therapeutic clearance efficiency depends on intervention administration time	[70]
	/	MVs activate ADP receptor P2Y12 on platelets, impairing aggregation and function	Detrimental	Coagulopathy risk rises prominently in severe acute TBI	[67]
Apoptotic Bodies	/	Apoptotic bodies found in thalamus and hippocampus 3 days post-TBI	Context-dependent	Timely phagocytic clearance relieves injury; impaired clearance triggers persistent inflammation	[83]

**Table 3:** Engineered extracellular vesicles for traumatic brain injury: therapeutic potential and translational barriers.

Therapeutic Potential (Engineered Strategies and Details)	References	Main Barriers to Clinical Translation (Challenges and Descriptions)	References
Endogenous modification Genetic engineering of parent cells to display brain-targeting ligands (e.g., RVG-Lamp2b, Angiopep-2) for enhanced BBB penetration.	[120,121]	Manufacturing variability Batch-to-batch heterogeneity, lack of standardized isolation/characterization protocols, and GMP-compatible scalable production challenges.	[145,146,147,148]
Exogenous cargo loading Physical methods (electroporation, sonication, extrusion, freeze–thaw) to encapsulate therapeutic molecules such as miR-124-3p, SOD, catalase, and small-molecule drugs.	[122,123,124,125]	Safety and dosing Tumorigenic potential, immunogenicity of allogeneic EVs, and ambiguous dose definition since particle number does not reflect bioactivity.	[153,154,155]
Surface engineering Chemical conjugation (click chemistry) or display of homing peptides/antibodies (e.g., RVG, anti-CD63) to achieve cell-type-specific targeting and reduce off-target effects.	[127,128,129,130,131]	In vivo tracking and stability Radioactive labeling may alter native EV behavior; freeze–thaw damage and lack of optimized lyophilization affect shelf life and potency.	[143,144,149,150,151]
Subtype-specific isolation Immunomagnetic nanopore sorting (TENPO chip) and immuno-affinity chromatography enable high-specificity enrichment of beneficial EVs or depletion of pathological subtypes (e.g., tau-bearing EVs).	[133,134,135,136,137,138]	Regulatory barriers No approved EV-based therapy exists; clear regulatory and harmonized SOP pathways are lacking.	[141,142,154]
Interdisciplinary innovations Single-EV transcriptomics and AI/ML approaches are employed to decipher cargo-function correlations and optimize drug-loading formulations.	[156,157,158]	Translational knowledge gaps Unclear mechanisms governing functional switching of EV subtypes, interspecies differences in BBB penetration, and immature integration of discovery tools into clinical pipelines.	[29,156]

## Data Availability

No new data were created or analyzed in this study. Data sharing is not applicable to this article.

## Abbreviations

ADEVs, astrocyte-derived extracellular vesicles; ApoBDs, apoptotic bodies; BBB, blood–brain barrier; BMSCs, bone marrow mesenchymal stem cells; CCL2, chemokine (C-C motif) ligand 2; CNS, central nervous system; CSF-EVs, cerebrospinal fluid-derived EVs; DAMPs, damage-associated molecular patterns; ESCRT, endosomal sorting complex required for transport; EVs, extracellular vesicles; FDA, Food and Drug Administration; FRET, fluorescence resonance energy transfer; hUCMSCs, human umbilical cord mesenchymal stem cells; ICAM-1, intercellular adhesion molecule-1; IL-1β, interleukin-1β; ILVs, intraluminal vesicles; MGEVs, microglia-derived extracellular vesicles; miRNAs, microRNAs; MVBs, multivesicular bodies; MVs, microvesicles; NDEVs, neuron-derived extracellular vesicles; NSCs, neural stem cells; PS, phosphatidylserine; SOD, superoxide dismutase; TBI, traumatic brain injury; TENPO, trajectory-etched magnetic nanopore; VWF, von Willebrand factor.
